# Genomic alteration in hereditary colorectal patients without mutations in mismatch repair genes

**DOI:** 10.1186/1753-6561-7-S2-O5

**Published:** 2013-04-04

**Authors:** Rolando AR Villacis, Érika MM Santos, Benedito M Rossi, Dirce M Carraro, Luiz P Kowalski, Silvia R Rogatto

**Affiliations:** 1 CIPE - AC Camargo Cancer Hospital, São Paulo, SP, Brazil; 2 Department of Pelvic Surgery, AC Camargo Cancer Hospital, São Paulo, SP, Brazil; 3 Department of Oncogenetic, Barretos Cancer Hospital, Barretos, SP, Brazil; 4 Department of Head and Neck Surgery and Otorhinolaryngology, AC Camargo Cancer Hospital, São Paulo, SP, Brazil; 5 Department of Urology, Faculty of Medicine, UNESP, Botucatu, SP, Brazil

## Background

Lynch Syndrome (LS) is the most common hereditary syndrome of colorectal cancer (CRC), caused by mutations in mismatch repair (MMR) genes. It is estimated that 50% of families classified according Amsterdam criteria not show germline mutations in MMR genes. These findings suggest that other genetic or epigenetic factors are associated with predisposition to CRC.

## Materials and methods

It was evaluated germline copy number variations (CNVs) in 57 patients with LS (Amsterdam Criteria), but without pathogenic mutations in MMR genes, by array CGH using the 4x180K platform (Agilent Technologies). Genomic data were extracted with Feature Extraction software and analyzed using Genomic Workbench software, statistical algorithm ADM-2 and threshold of 6.7.

## Results

It was found 252 CNVs (4.4 ± 3.6 CNVs/individual), including 104 genomic gains and 148 losses. After comparison with a reference group, composed of 100 healthy Brazilian women (Krepischi et al., 2012) and the Database of Genomic Variants (DGV-hg18), 106 rare CNVs were identified in 41 cases and 10 new rare CNVs in six cases. Four rare CNVs, of the same size, were detected in at least three cases: 1q21.1, 7p22.3, 11q13.2 and 15q11.2. Four patients had new rare CNVs mapped at 7p22.3. In 7p22.3 and 15q11.2.

## Conclusions

Putative candidate genes mapped on 7p22 are suggestive to be associated with hereditary predisposition to CRC. The relatives of those probands are being evaluated to confirm the segregation of the most important alterations and their association with CCR predisposition.

## Financial support

FAPESP and CAPES.

